# A hormone-related female anti-aphrodisiac signals temporary infertility and causes sexual abstinence to synchronize parental care

**DOI:** 10.1038/ncomms11035

**Published:** 2016-03-22

**Authors:** Katharina C. Engel, Johannes Stökl, Rebecca Schweizer, Heiko Vogel, Manfred Ayasse, Joachim Ruther, Sandra Steiger

**Affiliations:** 1 Institute of Evolutionary Ecology and Conservation Genomics, University of Ulm, 89081 Ulm, Germany; 2 Institute of Zoology, University of Regensburg, 93053 Regensburg, Germany; 3 Department of Entomology, Max Planck Institute for Chemical Ecology, 07745 Jena, Germany

## Abstract

The high energetic demand of parental care requires parents to direct their resources towards the support of existing offspring rather than investing into the production of additional young. However, how such a resource flow is channelled appropriately is poorly understood. In this study, we provide the first comprehensive analysis of the physiological mechanisms coordinating parental and mating effort in an insect exhibiting biparental care. We show a hormone-mediated infertility in female burying beetles during the time the current offspring is needy and report that this temporary infertility is communicated via a pheromone to the male partner, where it inhibits copulation. A shared pathway of hormone and pheromone system ensures the reliability of the anti-aphrodisiac. Female infertility and male sexual abstinence provide for the concerted investment of parental resources into the existing developing young. Our study thus contributes to our deeper understanding of the mechanisms underlying adaptive parental decisions.

## Abstract (web summary)

Parents are faced with the dilemma whether to invest in their current offspring, or potential future young. Here, Engel *et al*. show that nutritionally-dependent young induce temporary infertility in female burying beetles, which in turn is signalled to the male partner via a hormone-related anti-aphrodisiac.

## Graphical abstract

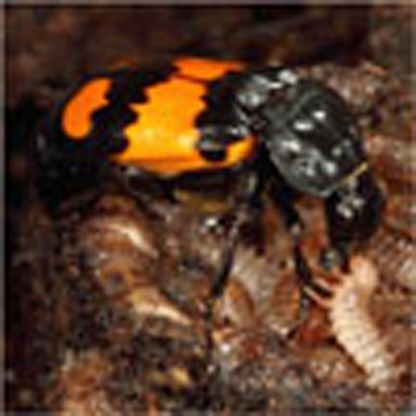

Family life creates a venue for cooperation and conflict^1^,^2^. Parents of many species cooperate to rear offspring, but nevertheless there can be intense conflicts between males and females over mating rate or how much each sex should invest in raising the young. Moreover, due to the high energetic and temporal costs of parental care, parents are ultimately faced with the reproductive dilemma of whether to continue to invest into the current offspring or to produce additional ones^3^. In general, parental care, such as offspring guarding or provisioning increases offspring lifetime reproductive success and thus can enhance parental fitness^4^. Consequently, as long as the benefits to the parents outweigh the costs of parental care, the decision should be made in favour of the current brood. However, the proximate mechanisms regulating resource flow into the appropriate direction are poorly understood. Especially, in mothers that provision their offspring with food, mechanisms should exist that direct nutrients and energy either into feeding the offspring or the development of new eggs/foetuses. Moreover, the entire family might benefit from parents synchronizing their sexual activity and only investing in costly matings when new or additional offspring are to be produced but not during the period of intensive parental care. However, coordination of parental and mating effort requires effective communication systems as the female needs to inform her male partner about whether she intends to reproduce or care. Although cues and signals of female receptivity or fertility are known to exist in some animal species^5^, our current knowledge of whether and how sexual activity and parental care is synchronized between partners is very limited. Mammals are one animal taxon where at least some information about the regulation of the resource trade-off within females as well as coordination of sexual activity between individuals is available. In many mammalian species, including humans, mothers are characterized by a postnatal infertility during the time of lactation^6^. The hormone prolactin secreted during lactation appears to be one factor suppressing ovulation^7^,^8^. Consequently, nutritional resources are allocated towards the existing young as long as they are needed, instead of developing new foetuses. In addition, many female mammals produce visual (for example, changes in colouration and/or swelling of the skin of the external genitalia) or chemical signals indicating the time of fertility^9^,^10^. These signals might help to synchronize mating activities, but also other functions have been discussed, especially as these signals often do not reveal the exact time of ovulation and do not necessarily coincide with the fertile phase^11^.

In insects, parental care including offspring provisioning has evolved many times^12^, but it is unknown how the conflicting energetic and nutritional demand of a current brood and sexual activity, including the development of new eggs, is balanced. To shed light on the physiological basis of parental investment strategies as well as on how sexual activity and parental care is coordinated between breeding partners, we studied burying beetles. These beetles are a prime example of insects performing elaborate biparental care^13^,^14^,^15^,^16^ and are known for their complex recognition and communication systems^17^. Burying beetles reproduce on small dead vertebrates and feed their developing young with pre-digested carrion food. The larvae of the burying beetle *Nicrophorus vespilloides* beg for food until about 3 days after hatching^18^. Afterwards they continue to feed independently on the carrion. The benefits of parental care in *N*. *vespilloides* are substantial. Not only larval survival rate but also larval mass at dispersal are significantly increased in broods receiving parental care compared with broods without parental attendance^19^. Particularly larval mass at dispersal is a measure of offspring quality, because it is highly correlated with adult body size which in turn enhances the effectiveness of securing a carcass needed for reproduction^20^,^21^. Hence, mothers are predicted to allocate their energy and nutritional resources into their existing larvae as long as they are needy instead of investing into the production of new eggs. Also time- and energy-consuming sexual activity distracting both parents from caring for their offspring should be avoided during this crucial time period of offspring feeding. Reliably communicating the reproductive status of the female to the male partner would therefore be advantageous for both parents and offspring.

The objectives of the current study were to investigate (1) how the resource trade-off between caring for current offspring and producing additional ones is regulated in females, (2) whether females signal their reproductive state to facilitate the coordination of sexual activity and parental care and (3) how the signal is reliably linked to fertility. Our first experiments addressed the question whether the fertility of female burying beetles is—similar to mammals—depressed during the time of offspring feeding and if this transient infertility is mediated by a hormone. Therefore, we determined egg production, hormone profiles and gene expression pattern of offspring provisioning and non-feeding females and manipulated the females' hormone titres experimentally. We demonstrate that females' fertility is indeed temporarily depressed when caring for nutritionally dependent larvae and this infertility is directly or indirectly linked to juvenile hormone (JH). In a previous study Engel *et al*.^22^ have shown that during the time of intensive offspring tending males desist from mating with their female partner. This observation and the finding that breeding females release a volatile chemical^23^ led us to the hypothesis that females produce a chemical signal linked to hormone production to coordinate sexual activity during periods of parental care. By conducting quantitative chemical analyses, we show that the emission of the volatile methyl geranate coincides with the presence of nutritionally dependent larvae and high JH levels. We further reveal that methyl geranate and JH production are linked via a shared pathway implying that females advertise their hormonal and therefore reproductive state. Finally, by means of electrophysiology and behavioural assays we demonstrate that methyl geranate serves as an antiaphrodisiac inhibiting male mating attempts. Overall, our results uncover mechanisms underlying parental care decisions, and illustrate how a physiological interplay between hormone and pheromone systems guarantees that both parents draw their attention towards the existing young as long as they are needy.

## Results

### Egg production and JH III during parental care

**Figure 1:**
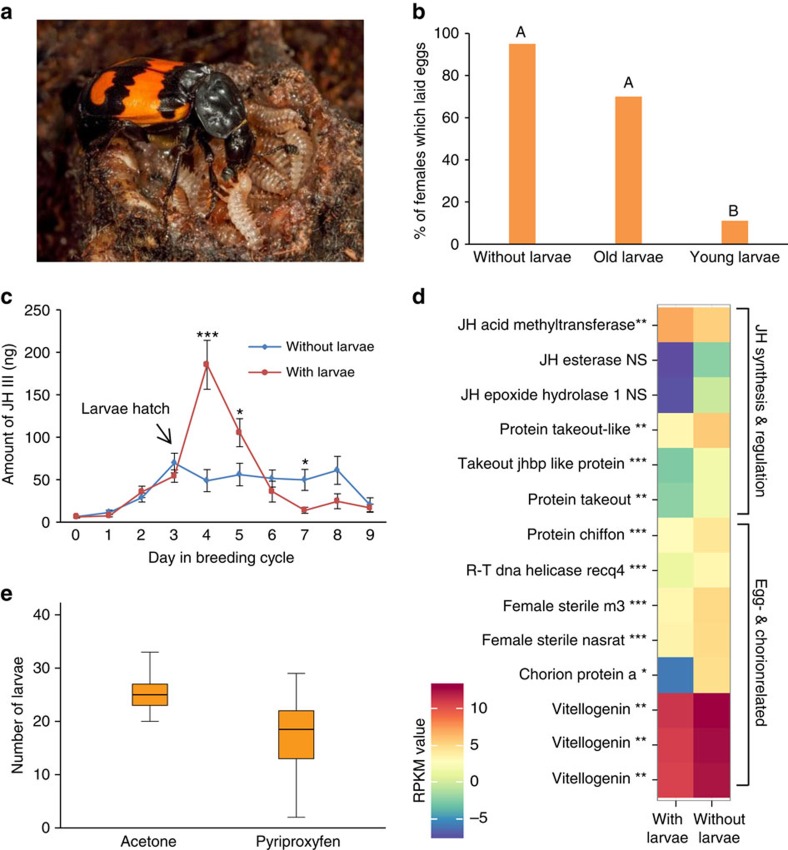
Offspring production and JH III. (**a**) Female *N. vespilloides* feeding a larva (reproduced with permission from the author, Heiko Bellmann). (**b**) Percentage of mothers laying eggs. Mothers were either denied access to their larvae after hatching (without larvae) or were caring for young needy larvae (young larvae) or older nutritionally independent larvae (old larvae). Different letters represent significant differences between groups at *P*<0.05 (*χ*^2^-test following Benjamini–Hochberg; *N*=18-20). (**c**) JH III profile in ng per individual (mean±s.e.) over an entire breeding cycle. Females were either allowed to care for their larvae (with larvae; *N*=191) or withheld from their hatched larvae (without larvae; *N*=186). There was an interaction effect of treatment group and day (Gaussian GLM: *F*_9,357_=9.91, *P*<0.0001). Note: on day 3 larvae hatched, but had not yet arrived on the carcass. (*P<0.05, **P<0.01, ***P<0.001). (**d**) Expression patterns of genes (heatmap based on geometric means) related to JH III biosynthesis/regulation and ovarian activity of females caring for larvae (day 4 in the breeding cycle) and females who were withheld from their larvae on hatching (day 4 in the breeding cycle; for each group *N*=3). Transcript abundances are expressed as log2 transformed normalized values (RPKM). (Student's *t*-test following Benjamini–Hochberg; *P<0.05, **P<0.01, ***P<0.001). (**e**) Number of larvae produced by females treated with pyriproxyfen or acetone (control). Boxplots show median, interquartile range, minimum/maximum range; *N*=19, Poisson GLM: Wald-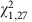
=18.34, *P*<0.0001.

To investigate whether oviposition is suppressed during provisioning of young, we provided females under three different parenting conditions with a new carcass to determine whether egg laying can be triggered. Females of the first treatment group (without larvae) had been allowed to lay eggs, but then were denied access to their larvae before they were provided with a new carcass (see Methods). Consequently, these females represented mothers that did not engage in offspring provisioning. Females of the second treatment group (old larvae) were caring for old, and already nutritionally independent larvae (4 days after hatching), when given a new carcass. Although these females had larvae, they also represented non-feeding mothers. Females of the third treatment group (young larvae) were caring for nutritionally dependent larvae (1 day after hatching; Fig. 1a) when a new carcass was provided. Hence, those females represented offspring provisioning mothers. Consistent with our hypothesis, mothers who were denied access to their larvae as well as mothers caring for older and already nutritionally independent larvae responded to the new carcass with egg laying (Fig. 1b). In contrast, in the majority of mothers with access to their newly hatched and therefore needy larvae oviposition was significantly suppressed (*χ*^2^-test, *N*=58, 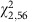
=29.1, *P*<0.0001, Fig. 1b).

Juvenile hormone III (JH III), a multifunctional hormone of insects, is well known to regulate female fertility by controlling the biosynthesis of vitellogenin (Vg) and its uptake by the growing oocyte^24^. JH III has been found to be elevated during parental care in the nearctic burying beetle *N. orbicollis*^15^,^25^, but its function remained unclear. In ants^26^,^27^, wasps^28^ and bees^29^ very high titres of JH or its analogues repress ovarian activity. We thus hypothesized that high JH III levels are associated with offspring feeding as well as suppression of ovarian activity and offspring production in *N. vespilloides* females. We indeed found that the JH III titres of mothers increased to very high levels directly after the hatching of their larvae (Fig. 1c; Supplementary Fig. 1). Corresponding to previous findings in *N. orbicollis*^30^, JH III peaked when offspring begging and provisioning is known to be most intense (24 h after hatching, day 4 in the breeding cycle)^18^,^31^, while it declined to pre-hatching levels when larvae can feed independently from the carrion resource (72 h after hatching, day 6 in the breeding cycle)^18^. In contrast, mothers whose larvae hatched but were experimentally prevented from accessing their offspring had significantly lower JH III titres and never reached the high levels of provisioning mothers (Fig. 1c; Supplementary Fig. 1). Sixty-six per cent of these females resumed egg laying, in contrast to zero per cent of offspring provisioning mothers. To verify the link between JH III and ovarian activity, we analysed the expression pattern of genes associated with JH biosynthesis/regulation and ovarian activity. Consistent with our prediction, offspring-provisioning mothers showed significantly higher expression of genes associated with JH III synthesis but lower expression of genes involved in JH degradation and egg production than non-provisioning mothers, whose larvae were withheld (Fig. 1d). Moreover, when females were treated with pyriproxyfen (PPN), a potent JH analogue^29^, reproduction was significantly reduced, and females treated with PPN produced fewer offspring than control females (Fig. 1e). Consequently, our results strongly suggest that needy larvae stimulate high JH levels in brood-caring mothers, which directly or indirectly lead to oviposition suppression.

### Linking hormone and pheromone production

**Figure 2:**
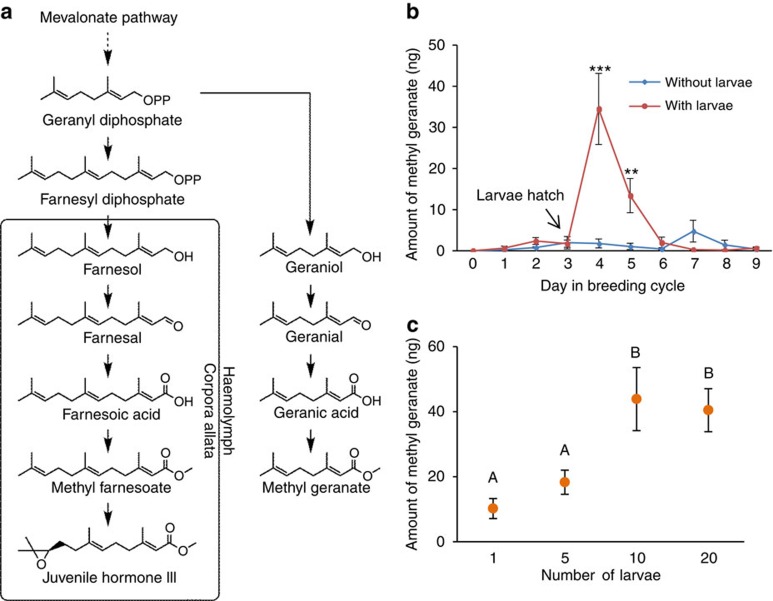
Methyl geranate biosynthesis and emission. (**a**) Putative biosynthesis of methyl geranate and its link to JH III. (**b**) Methyl geranate emission in ng per individual per 20 min (mean±s.e.) over an entire breeding cycle. Females were either allowed to care for their larvae (with larvae; *N*=170) or withheld from their hatched larvae (without larvae; *N*=169). There was an interaction effect of treatment group and day (Gaussian GLM: *F*_9,319_=11.35, *P*<0.0001). Note: on day 3 larvae hatched, but had not yet arrived on the carcass. (*P<0.05, **P<0.01, ***P<0.001). (**c**) Amount of methyl geranate (mean±s.e.) emitted over a period of 20 min by mothers caring for 1, 5, 10 or 20 larvae. Different letters represent significant differences (Gaussian GLM following Benjamini–Hochberg; *N*=20–30).

During the time when the offspring are still needy, not only egg production but also costly male mating attempts distracting both parents from caring for offspring should be avoided. Copulations, for example, might divert the attention from protecting the offspring against enemies and restrain from offspring feeding. Therefore, reliably signalling their hormonal state to the male should be advantageous for females. Fathers in turn should respond to the signal by ceasing sexual activities, as they do not gain any benefits from copulating during the female's period of infertility. An excellent candidate for such a reliable signal is methyl geranate, a volatile that has been shown to be emitted by breeding females but has been studied hitherto only in the context of partner recognition^23^. Methyl geranate is structurally related to JH III (Fig. 2a) and has been hypothesized to share the same biosynthetic pathway as the hormone, although experimental evidence is missing^23^. To test for a functional linkage of the hormone system and methyl geranate biosynthesis, we injected deuterium-labelled geranyl pyrophosphate^32^, a known precursor of JH III in insects^33^ and putative key component in the postulated shared pathway (Fig. 2a), into offspring-provisioning females. Subsequent headspace analyses revealed that injected females emitted deuterium-labelled methyl geranate (Supplementary Fig. 2). Consequently, the volatile substance is indeed a suitable candidate for reliably reflecting the female's hormone level and therefore her reproductive state, as it shares the same metabolic pathway with the hormone. In fact, when measuring JH III levels and released methyl geranate quantities of females during an entire breeding cycle, we found a strong correlation between the hormone and the volatile substance (Pearson correlation, *N*=332, *r*=0.5, *P*<0.0001). In females caring for larvae, methyl geranate emission tightly followed JH levels and peaked along with the hormone titre at the time when offspring begging and feeding is known to be most intense (Fig. 2b). In contrast, mothers who were denied access to their larvae emitted little or no methyl geranate (Fig. 2b). Furthermore, methyl geranate emission closely matched the observed mating pattern during a breeding cycle^22^. Males discontinue mating with mothers during the time of offspring provisioning but do not stop mating with females whose larvae had been withheld and therefore resume egg laying^22^. To further confirm that methyl geranate reliably reflects the female's reproductive state, we exploited the fact that females are less likely to resume egg laying the more offspring they have to care for. An earlier study found that one larva prevented about 20% of the mothers from producing a new clutch, and more larvae prevented a significantly higher percentage^34^. Consequently, we manipulated the amount of larvae a female had to care for and quantified methyl geranate emission. Indeed, methyl geranate quantity reliably reflected oviposition probability: the more larvae the mother had to care for, the higher was the amount of methyl geranate emitted (Gaussian GLM, *N*=105, *F*_3,101_=7.50, *P*<0.001; Fig. 2c). Furthermore, those mothers that resumed egg laying were characterized by a lower JH III titre than mothers that did not engage in oviposition (*N*=103, mean±s.e., egg-laying mothers: 84.52±8.91 ng, non-egg-laying mothers: 122.17±8.63 ng; Gaussian GLM, *F*_1,101_=4.29, *P*=0.04).

### Function of methyl geranate as anti-aphrodisiac

**Figure 3:**
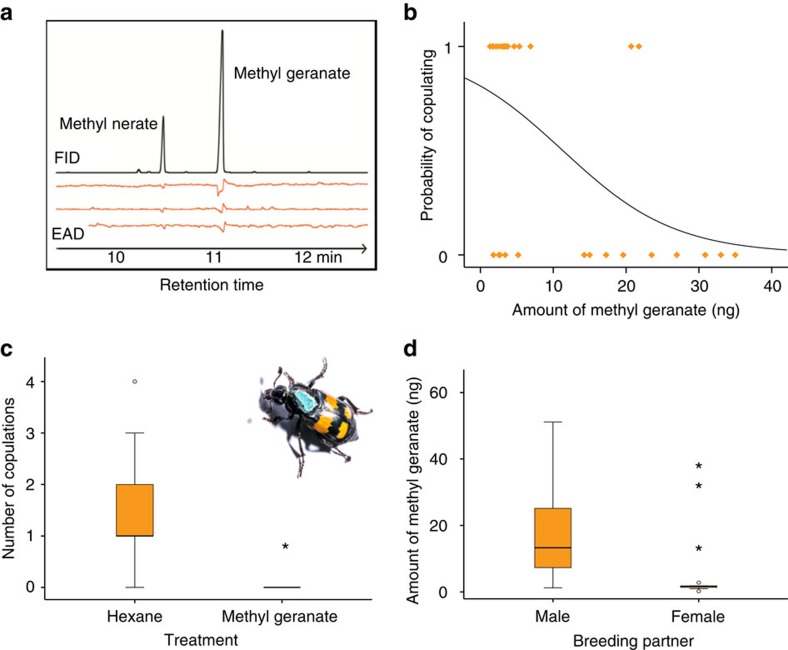
Function of methyl geranate as an anti-aphrodisiac. (**a**) GC-EAD chromatogram of synthetic methyl geranate using antennae of male *N. vespilloides*. All three male antennae responded to methyl geranate, and to methyl nerate that occurred as a byproduct in the sample. (**b**) The link between methyl geranate quantities released by females over a period 20 min and male copulation probability. Symbols represent original data. Curve represents the calculated logistic regression. *N*=31, Wald-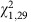
=6.41, *P*=0.01. (**c**) Number of copulations per female. Females were equipped with a septum (see picture) that was either treated with a methyl geranate solution or pure hexane (control). Boxplots show median, interquartile range, minimum/maximum range excluding outliers, outliers (circle, >1.5 × interquartile range) and extreme outliers (asterisks, >3 × interquartile range). *N*=27, Poisson GLM: Wald-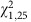
=9.2, *P*=0.002. Image of the beetle reproduced with permission from the author, Kai Schillinger. (**d**) Amount of methyl geranate emitted by females over a period of 20 min breeding with a male or a female partner. Boxplot conventions as in (**c**). *N*=28, Gaussian GLM: *F*_1,26_= 5.78; *P*=0.02.

Having established that methyl geranate reliably reflects hormone level and consequently the reproductive state of females, we further evaluated its putative function as an anti-aphrodisiac, a chemical substance that inhibits copulation^35^,^36^. Using gas chromatography coupled with electroantennographic detection (GC-EAD), we found that male antennae respond to synthetic methyl geranate (Fig. 3a). Furthermore, we observed the copulation behaviour of pairs of breeding *N. vespilloides* with and without larvae for 90 min and quantified the methyl geranate emitted by the females. Consistent with our expectation, we found that the more methyl geranate was emitted by a female, the lower was the probability that a male copulated with her (Fig. 3b). In addition, the number of copulations performed was dependent on the amount of methyl geranate emitted and females with lower amounts of methyl geranate received more copulations than females with higher amounts of methyl geranate (Poisson GLM, *N*=31, Wald-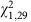
=7.7, *P*=0.006; Supplementary Fig. 3). To verify the function of methyl geranate as an anti-aphrodisiac, we established a bioassay in which females were equipped with a septum emitting synthetic methyl geranate (430 ng±57.8 ng per 20 min; corresponds to four female equivalents based on the maximum measured emission) fixed on their pronotum (Fig. 3c). Control females also carried a septum that was treated with the pure solvent. In agreement with our prediction, male copulation activity was significantly decreased by methyl geranate emission (methyl geranate: 23.08% copulating males, solvent control: 78.57% copulating males; *χ*^2^-test, *N*=27, 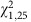
=8.3, *P*=0.004; number of copulations; Fig. 3c).

Two female burying beetles attracted to the same carcass usually fight over its possession. On relatively large carcasses, however, joint breeding can occur and the females cooperate in providing parental care^37^. To corroborate that the primary function of methyl geranate is to deter males from copulating, we quantified methyl geranate released by mothers in female/female and female/male couples breeding on a large carcass. In fact, the sex of the breeding partner had a significant effect on methyl geranate emission. When breeding with a male partner, females emitted significantly higher amounts of methyl geranate than when breeding with a female (Fig. 3d). Consequently, this result confirms the idea that methyl geranate specifically evolved to function as an anti-aphrodisiac.

## Discussion

In insects, post-hatching parental care has evolved independently in at least 10 different orders^38^, but little is known about the physiological mechanisms underlying parental care and intra-familial interactions. Here, we specifically addressed fundamental questions about how the trade-off between care for current offspring and the production of new eggs is physiologically regulated and how mating and parental effort is synchronized between breeding partners.

Our first key finding was that oviposition is suppressed when offspring fitness crucially depends on parental feeding, but that females resume egg laying if they have no access to their larvae or if larvae are already nutritionally independent. Furthermore, hormonal profiles, expression pattern of genes involved in hormone biosynthesis and egg production, and the result of hormonal treatment strongly suggest that JH III is the physiological key factor responsible for the reported temporary suppression of fertility and for the maintenance of parental care behaviour. The exact mode of action of JH III is not yet known, but we suggest two possible mechanisms: In many female insects, JH appears to be a critical factor modulating Vg biosynthesis in the fat body and its uptake by the growing oocytes. Although JH III has often been found to function as gonadotropic hormone stimulating Vg production^39^,^40^, there are also examples where very high titres supress Vg biosynthesis^28^,^29^. The fact that in our study Vg genes were significantly downregulated at the same time as JH III levels were drastically increased, but upregulated at lower JH levels, supports this hypothesis. An alternative explanation is that JH III indirectly represses egg production by diverting energy and nutrients away from reproduction and towards parental care, especially towards predigestion of food and its regurgitation to offspring. Such a novel role of JH III in meeting the high energy demands of parental care has been previously suggested by several authors^25^,^41^,^42^. However, irrespective of whether JH directly or indirectly causes a suppression of fertility, the outcome is the same: energy and nutrients which would otherwise have been invested in egg development are now available for tending the current offspring. Such a hormonally-induced infertility during the time of offspring feeding is strongly reminiscent of the lactational amenorrhea in female mammals. Here, the hormone prolactin appears to simultaneously stimulate milk production and parental behaviour in mothers and to inhibit ovulation^7^,^8^. Considering these parallels between mammals and social beetles, we might hypothesize that a hormone-mediated transient infertility is a general scheme in non-seasonal breeding animals that feed their offspring.

Due to the fact that we carefully synchronized and standardized all the broods, we could show that it is the young larvae that trigger the high rise in JH levels. This finding is also supported by an earlier study in *N. orbicollis*, where a continuous supply of first instar larvae led to a prolonged period of high JH titre^25^. Currently, we can only speculate about the exact nature of the stimulus that prompts JH production. In mammals, including humans, it appears that it is the frequency of the suckling stimulus of the pups/babies that induces and maintains prolactin secretion^43^,^44^,^45^. In burying beetles, larvae show a specialized begging behaviour, in which they rear up and wave their legs, thereby touching the parents' mouthpart to obtain food^46^,^47^. Consequently, these mechanical cues might trigger JH III production and release. Interestingly, studies on honey bees have shown that the pheromone (*E*)-β-ocimene, produced by young larvae, not only accelerates the onset of foraging behaviour in workers^48^,^49^, but also inhibits the workers' ovary development^50^. Therefore, we suggest that future studies should compare the chemicals released by young and old burying beetle larvae, as substances might be involved that either act as an honest signal of hunger state or as a primer pheromone inducing JH production and therefore a repressed fertility in mothers. Certainly, if a primer pheromone is involved that directly affects the mother's JH titre, offspring may gain substantial control over maternal care, influencing the mothers' trade-off between current and future reproduction^51^. Irrespective of the nature of the stimulus, our data also indicate that the response of the mother is threshold dependent, as one larva is sometimes not enough to trigger sufficient JH III to suppress egg production (see also ref. 34). Furthermore, our findings shed new light on the infanticide committed by male burying beetles after having taken over the brood chamber of a resident couple^52^. By killing the offspring sired by the previous male, the intruder hastens his own reproduction, given that the resident mother would not resume production of new eggs as long as the stimulus coming from nutritionally dependent larvae is present. Again, this adaptation finds its parallels in mammals^53^.

The second key finding of our study was that females communicate their JH titre, and therefore their reproductive state, to their male partner, thereby influencing male social behaviour during parental care. In general, fertility^5^,^54^,^55^,^56^,^57^ or infertility signals^58^,^59^,^60^ are common among females across animal taxa, from insects to reptiles and mammals, but for the vast majority of species the physiological processes linking fertility/infertility and the communicational signal are unknown. An exception are some teleost fish, in which it has been shown that steroid hormones or derivatives of the hormones responsible for oocyte maturation act as sex pheromones, inducing milt production in males^61^,^62^. However, in contrast to aquatic environments where high-molecular-weight substances such as numerous hormones can directly function as attractants, in terrestrial environments pheromone molecules must normally be volatile and therefore of lower molecular mass. Here we report the mechanism explaining the link between fertility and a chemical signal in a brood-caring beetle. To the best of our knowledge, this is the first time such a linkage has been found in an insect. Using a stable isotope-labelling experiment, we revealed that the pheromone methyl geranate derives from the same biosynthetic pathway as the hormone modulating fertility. The shared pathway therefore provides an explanation for how the reliability of the communication system is maintained, as the pheromone can only be produced if the mevalonate pathway, the fundamental pathway of hormone biosynthesis, runs at a high level. This has further implications for our understanding of the evolution of communication systems. Within the framework of sexual selection, it has been previously suggested that one way of how condition-dependent ornament expression is linked to an animal's condition is by a shared pathway of ornament production and vital biochemical processes^63^.

In a previous study, we have found that male *N. vespilloides* copulate frequently in the beginning of a breeding attempt, stop mating as soon as young larvae are present, but resume mating when larvae are nutritionally independent^22^. Furthermore, males never cease to mate if larvae do not arrive at the carcass to be fed^22^. Our current study unravels the mechanism underlying the observed male mating behaviour: the female-produced methyl geranate acts as anti-aphrodisiac, inhibiting male copulation behaviour. The methyl geranate emission profile of breeding females matches the males' mating behaviour remarkably well and our behavioural assay using synthetic methyl geranate conclusively shows that males respond to the volatile by becoming sexually inactive. Overall, empirical and theoretical considerations suggest that female fitness is maximized at a lower mating rate than male fitness, giving rise to a sexual conflict over mating rate^64^. For female burying beetles, about two mating events are sufficient to fertilize all their eggs, and none of the many experiments conducted so far could find any benefits for females of repeated or multiple mating^65^,^66^. In view of this, and of the fact that studies found substantial costs of mating to females in a wide range of animal groups^67^, we can assume that the high mating rate of *N. vespilloides* in the beginning of a reproductive bout is not in the interest of the female^68^. Such a sexual conflict over mating rate would also exist during the periods of offspring feeding, but due to the temporary infertility of the female there is a sudden concurrence of interests between the breeding partners. However, only thanks to the evolution of a reliable signal of reproductive state could the common interests of both parents be realized. We believe that methyl geranate is an important component of family life in burying beetles, as it allows the coordination of sexual activity and parental care. As mating consumes time and energy and distracts from feeding and defending the young, these chemical signal also benefits the offspring.

Our final experiment with the two jointly breeding females corroborates our hypothesis that the primary function of methyl geranate is to deter male copulations, as it is only released in high quantities by mothers breeding with a male but not with a female partner. Previous studies have demonstrated that male and female burying beetles can recognize their breeding partner and distinguish them from non-breeding infanticidal intruders^69^,^70^. Methyl geranate is one chemical component of the odour bouquet males use for partner recognition^23^. However, Steiger^17^ already hypothesized that it is unlikely that this is the main function of methyl geranate, as it is only emitted by females, while the partner recognition mechanisms work reciprocally for both sexes. On the basis of the results of the present study, we conclude that the signal evolved as an anti-aphrodisiac. Because breeding females release methyl geranate at a certain point of their breeding cycle, it is—during the process of template updating—incorporated into the male's recognition template used for discriminating between their own partner and female intruders. Finally, the fact that females can adjust their methyl geranate emission according to their social environment irrespective of their own physiological condition suggests that the volatile is not an inadvertent by-product of juvenile hormone synthesis, but rather that its production and/or release can be switched on and off adaptively. This does not compromise the reliability of the signal, as the prerequisite of pheromone production is an active mevalonate pathway, which might necessarily lead to a high JH titre.

In conclusion, our study allows novel insights into the physiological mechanisms underlying parental decisions. Similar to non-seasonal mammals, a physiological interplay between the hormone and communication systems guarantees that parents do not invest in costly sexual activity or the development of new offspring as long as the existing young are nutritionally dependent. However, in contrast to many mammalian species, where females appear to communicate periods of fertility, that is, the time close to ovulation, burying beetles broadcast their temporary infertility during the period of intensive parental care. The emission of an anti-aphrodisiac reliably reflecting a female's hormonal state facilitates a synchronization of mating events between the sexes and ensures that parental attention is directed towards the developing young.

## Methods

### Insects

Experimental *N. vespilloides* animals were the first- to fourth-generation offspring of beetles collected from carrion-baited pitfall traps in a deciduous forest near the University of Ulm, Germany (48° 25′ N, 09° 57′ E). Beetles were maintained in temperature-controlled chambers at 20 °C with a 16:8 h light:dark cycle. Before the experiments, groups of up to five adults of the same sex and family were kept in small plastic containers (10 × 10 cm and 6 cm high) filled with moist peat and fed freshly decapitated mealworms (*Tenebrio molitor*) twice a week. At the time of experiments all experimental animals were virgin, between 20 and 40 days old and not related to each other within a specific treatment group of an experiment.

### General experimental design

All experimental beetles, unless otherwise stated, were handled as follows: non-related males and females were placed pairwise in plastic containers (10 × 10 × 6 cm) half-filled with moist peat and provided with a mouse carcass (10, 20 or >30 g, depending on the experiment; Mäu-Ra Farm, Radensleben, Germany). Once the carcass was buried, the containers were kept in darkness and all following manipulations were performed under red light. After 48 h the beetles and their carcass were transferred to a new container. The old container was provided with a small piece of mouse. As larvae crawl towards the carrion immediately after hatching, this procedure allowed us to determine the time when a pair's larvae began to hatch. Embryonic development takes, on average, 56 h at 20 °C (ref. 34). We first checked for the presence of larvae 64 h after the adults had been provided with a carcass and every 6 h thereafter. As soon as the first larva was observed, the parents were transferred to a new box and a standardized number of first-instar larvae were placed on or near the carcass to be fed by the adults. The standardization of larval number allowed us to control for effects of brood size on parental physiology or care behaviour^71^. All experiments were performed in temperature-controlled chambers at 20 °C.

### Oviposition and offspring provisioning

To test whether non-feeding mothers oviposit, whereas mothers caring for needy larvae do not engage in egg laying, we randomly assigned experimental females to three different treatment groups. All experimental females were provided with a male partner and a 10-g carcass, but after a specific time the old carcass was replaced by a fresh carcass. This was done to test whether a new carcass triggers oviposition or not. Females of one treatment group (old larvae) were allowed to care for 10 of their larvae for 4 days before we exchanged carcasses (the larvae remained with the female). At this point in their development, larvae are already nutritionally independent^18^. In the two other treatment groups, carcasses were exchanged after the larvae started to hatch, but only females of one group (young larvae) received 10 freshly hatched larvae to care for; such larvae are needy and are fed by the females. Females of the other group (without larvae) did not receive any larvae and therefore were not able to engage in offspring provisioning. After having exchanged the carcasses, females were left undisturbed for a specific amount of time to give them a chance to discover the new carcass and to produce new eggs. Females usually begin to oviposit about 12 h after carcass detection^72^ and the mean duration of egg-laying period is about 30 h (refs 34, 73). Forty-eight hours after carcass exchange, beetles along with the new carcass were transferred to new containers and the old containers were searched for eggs. This was done by scanning the peat of the old container manually, as the white eggs are easy to detect in the dark background of the peat. The procedure of transferring the beetles along with their carcasses to a new container and searching for new eggs was repeated a second time with any pairs that had not laid any eggs during the first period. We used a *χ*^2^-test to test for differences in oviposition events between treatment groups. Pairwise comparisons were corrected following the Benjamini–Hochberg procedure^74^. This and all further statistical analysis were done using SPSS 19 (IMB SPSS Statistics, Germany).

### JH III and methyl geranate profile during a breeding cycle

To gain insight into the pheromonal and hormonal profiles of the female during an entire breeding cycle (∼9 days), *N. vespilloides* females (*N*=387) were randomly assigned to two treatment groups (‘with larvae', ‘without larvae'), each consisting of 10 subgroups (see Supplementary Table 1 for sample size of each subgroup). Each subgroup represents a day in the breeding cycle (day 0–day 9). Experimental females were provided with a male partner and a 10-g mouse carcass. Pairs from the treatment group ‘with larvae' were allowed to care for 10 of their own and therefore related larvae; in the treatment group ‘without larvae', larvae were withheld from their parents to force females to resume egg laying. Each female was subjected once to volatile (‘headspace analysis', see below) and haemolymph collection (see below) at a pre-designated time. Females of the subgroup ‘day 0' were sampled before carcass introduction. The females of the next groups (‘day 1' and ‘day 2') were sampled and 48 h after they had been provided with a carcass. Females of the subgroups ‘day 3' were sampled at the time of larval hatching, which is not necessarily 72 h after carcass provisioning, as egg laying and consequently larval hatching does not occur in perfect synchrony between different breeding pairs. Females of all subsequent subgroups were sampled 24 h (day 4), 48 h (day 5) and so on, after the larvae of the respective female had hatched (for example, if larvae of a female of the subgroups ‘day 7' hatched at 06:00, sampling took place 96 h later at 06:00). We chose this non-repeated measurement design as it is very likely that haemolymph collection has an effect on subsequent female behaviour and physiology. Therefore, all beetles were freeze-killed after haemolymph collection.

After headspace sampling (see below), each female was weighed and all her haemolymph was collected. Haemolymph samples were obtained by piercing the intersegmental membrane between pro- and mesothorax with a fine cannula and aspirating the oozed haemolymph into a calibrated glass capillary. After quantifying the volume, the haemolymph was transferred to a 1.5-ml cone-shaped glass vial by blowing nitrogen through the capillary. An amount of 100 μl hexane containing 20 ng of vernolic acid methyl ester (Sigma-Aldrich, Deisenhofen, Germany) as an internal standard was added to the vial. The mixture was vortexed for 30 s and the clear hexane phase was transferred to a new vial, concentrated to a volume of 25 μl and subjected to size exclusion high performance liquid chromatography (SE-HPLC) for cleaning. SE-HPLC settings and all further steps for quantifying JH III, including gas chromatography-mass spectrometry analysis and calibration, were performed following the protocol described in ref. 75. We used Gaussian GLMs to test for effects of treatment and day on methyl geranate emission and JH level and a Pearson correlation to test for relationship between pheromone and hormone levels. As we measured the absolute amount of methyl geranate emitted by an individual, we also used the absolute amount of JH per individual for statistical analyses. However, as studies usually provide hormone titres, we additionally calculated JH titres (ng JH III per μl haemolymph; Supplementary Fig. 1). Furthermore, we calculated the proportion of females that resumed egg laying. Because females were freeze-killed subsequent to the treatment, we could only consider females whose larvae had already hatched (day 4 to day 9). To determine whether a female had resumed egg laying, we counted the number of larvae in the treatment group with larvae; in the treatment group without larvae, females were transferred every 48 h to a new box and the old box was checked daily for newly hatched larvae.

### RNA isolation

RNA was extracted from females that had been caring for 10 larvae for 24 h (corresponds to the treatment group ‘with larvae, day 4', Fig. 1b) and females whose larvae had hatched 24 h earlier, but were not allowed to care for their larvae (corresponds to the treatment group ‘without larvae, day 4'; Fig. 1b; each *N*=3). Individual beetles (a single female per biological replicate per treatment) were frozen in liquid nitrogen and ground with a mortar and pestle, and total RNA was extracted from a fraction of the powdered material, using TriReagent (Molecular Research Centre, Cincinnati, OH, USA) and further purified using the DirectZol Kit (Zyme Research) following the manufacturers' guidelines. The integrity of the RNA was verified using an Agilent 2,100 Bioanalyzer and a RNA 6,000 Nano Kit (Agilent Technologies, Palo Alto, CA). The quantity of RNA was determined using a Nanodrop ND-1,000 UV/Vis spectrophotometer (Thermo Scientific).

### Illumina sequencing

Transcriptome sequencing of each of the six RNA samples (two different female samples with three biological replicates each) was performed with RNA fragmented to an average of 150 nucleotides. Sequencing was carried out by the Max Planck Genome Centre Cologne (MPGCC) on an Illumina HiSeq2500 Genome Analyzer platform using paired-end (2 × 100 bp) read technology. This yielded ∼25–28 million reads for each of the six samples, respectively. Quality control measures, including the filtering of high-quality reads based on the score given in fastq files, removal of reads containing primer/adaptor sequences and trimming of read length, were carried out using CLC Genomics Workbench v6.5 (http://www.clcbio.com).

### Transcriptome assembly and annotation

The *de novo* transcriptome assembly was carried out with the same software, combining all of the six RNAseq samples, and selecting the presumed optimal consensus transcriptome as described in ref. 76. Any conflicts among the individual bases were resolved by choosing the base with highest frequency. Contigs shorter than 250 bp were removed from the final analysis. The resulting final *de novo* reference transcriptome assembly (backbone) of *N. vespilloides* contained 55,934 contigs with a N50 contig size of 954 bp and a maximum contig length of 18,454 bp. The transcriptome was annotated using BLAST, Gene Ontology and InterProScan searches using BLAST2GO PRO v2.6.1 (www.blast2go.de)^77^ as described in ref. 76.

### Digital gene expression analysis

Digital gene expression analysis was carried out by using QSeq Software (DNAStar Inc.) to remap the Illumina reads from all six samples onto the reference transcriptome and then counting the sequences to estimate expression levels, using previously described parameters for read mapping and normalization^76^. Biases in the sequence data sets and different transcript sizes were corrected using the RPKM algorithm (reads per kilobase of transcript per million mapped reads) to obtain correct estimates for relative expression levels. To control for the effect of global normalization using the RPKM method, we also analysed a number of highly conserved housekeeping genes, including several genes encoding ribosomal proteins (rpl3, rpl5, rpl9, rpl22e, rps3a, rps5, rps8, rps18 and rps27), elongation factor 1alpha and eukaryotic translation initiation factors 4 and 5. The overall expression levels across samples and treatments for these housekeeping genes was lower than 1.3-fold between samples (based on log2 transformed RPKM values), indicating they were not differentially expressed. Transcript abundances of genes of the different treatment groups were compared using a Student's *t*-test with multiple testing correction using FDR (Benjamini and Hochberg) as implemented in the QSeq Software.

### Pyriproxyfen treatment

PPN is a potent JH analogue, which mimics the effects of JH but is metabolically more stable. We applied 5 μg PPN (Sigma-Aldrich, Taufkirchen, Germany) dissolved in 2 μl acetone topically on the intersegmental membrane between pro- and mesothorax of female beetles. The control group was treated with 2 μl acetone only. Afterwards females were provided with a 10-g mouse carcass in a box filled with peat to trigger oviposition. After 24 h, the PPN/acetone procedure was repeated. Two days after carcass provisioning the parents were removed and newly hatched larvae counted until hatching ceased. The total amount of applied PPN was similar to the amount used in other studies^26^,^29^. To test whether the treatment (PPN versus control) had an effect on the number of offspring produced, we performed a Poisson GLM.

### Effect of brood size on methyl geranate emission

Pairs of *N. vespilloides* beetles were provided with a 20-g mouse carcass and allowed to care for 1, 5, 10 or 20 larvae, respectively. After caring for larvae for 24 h, the number of surviving larvae was counted and the females were subjected to headspace analyses. To be able to provide each experimental pair with the planned number of larvae, we additionally provided a large number of *N. vespilloides* pairs with a 10-g mouse carcass, and use these pairs as ‘larvae donors'^78^. A Gaussian GLM was performed with the number of larvae as fixed factor and the amount of methyl geranate as dependent variable.

### Deuterium labelling experiment

To test whether the production of JH III and methyl geranate is physiologically linked via the mevalonate pathway^33^ (Fig. 2), we injected a deuterium-labelled geranyl pyrophosphate precursor^32^,^79^ ((*E*)-3,7-dimethylocta-2,6-dien-1-yl-2-d diphosphate, [2-^2^H]-GPP, kindly provided by W. Boland, Max-Planck-Institute for Chemical Ecology, Jena, Germany; see Supplementary Fig. 2) into the body cavity of brood-caring *N. vespilloides* females. Geranyl pyrophosphate is a well-known metabolic precursor of JH III^33^. Pairs of beetles were provided with a 10-g mouse carcass and left undisturbed until we started to control for larval hatching. After the beetles had cared for their larvae for up to 13 h, the female was removed and subjected to [2-^2^H]-GPP injection. We injected 10 μg [2-^2^H]-GPP per female diluted in 1 μl distilled water either into the abdomen, into the intersegmental membrane between pro- and mesothorax, or into the ventral cervical membrane using a pointed glass microcapillary, which was mounted to a micromanipulator and connected to a model 2010 Nanoliter Injector (World Precision Instruments, Berlin, Germany). After injection, females were returned to their respective brood and male partner and were subjected to headspace analyses as described below between 2 and 6 h later. The incorporation of the deuterium-labelled geranyl pyrophosphate into methyl geranate was concluded from the increase in the abundance of diagnostic ions of methyl geranate^80^.

### Gas chromatography coupled with electroantennographic detection

The electrophysiological response of *N. vespilloides* male antennae towards methyl geranate was tested using GC-EAD. The same GC-EAD set-up as in ref. 81 was used, but the GC was equipped with a DB-5 capillary column (30 m × 0.25 mm i.d., 25 μm film thickness, J & W Scientific, Folsom, CA, USA) and the oven temperature was raised from 50 °C to 220 °C at a rate of 10 °C per min. The final temperature was held for 2 min. The responses of three male antennae were tested to 50 ng of synthetic methyl geranate (100 ng μl^−1^ pentane, 1 μl injected, Split FID:EAD=1:1, mixture of isomers, Santa Cruz Biothechnology, USA). The antennae were cut at their base and at the tip. The excised antenna was mounted between two glass electrodes filled with insect Ringer solution (see ref. 81 for further details).

### Copulation behaviour and natural methyl geranate emission

Mating behaviour of *N. vespilloides* males in two different breeding contexts was analysed by observing them continuously for 90 min. Experimental *N. vespilloides* pairs were provided with a 10-g mouse carcass and divided in two different treatment groups. In the first treatment group, pairs were allowed to care for 10 of their own larvae and in the second treatment group larvae were withheld from their parents. Observation took place 48 h after larval hatching. During observation we recorded whether they copulated or not, and if they did, how often they engaged in copulations. At the end of each observation period the females were directly subjected to headspace analyses to quantify methyl geranate emission. To test whether male copulation behaviour (yes/no) or the number of copulations was/were dependent on the amount of methyl geranate a female emitted, a logistic regression and a Poisson GLM was performed.

### Bioassay with synthetic methyl geranate

To test the effect of synthetic methyl geranate on male copulation behaviour, we used triangular shaped pieces (4 × 4 × 5 mm, 1 mm thick) of Supelco Molded Thermogreen LB-2 silicone septa (Sigma-Aldrich, Taufkirchen, Germany), which were either conditioned for 7 h in 1 ml *n*-hexane (control) or a solution of 10 mg synthetic methyl geranate diluted in 1 ml *n*-hexane. Subsequently the silicon pieces were left under a fume hood for 15 h to allow the solvent to evaporate. Headspace analyses (*N*=7) of the silicone pieces revealed that after this procedure they emitted a relatively constant amount of methyl geranate (430 ng±57.8 ng per 20 min) over a long time period. The silicon pieces were attached to females that were breeding with a male at a 10-g carcass for about 120 h, but were not allowed to care for larvae (corresponds to the treatment group ‘without larvae, day 5'. These females thus emitted no or only trace amounts of their own methyl geranate; Fig. 1d). After fixing the silicon pieces on a female's pronotum with superglue, the female and the male and their carcass were transferred to a small plastic container (7 × 3 × 3 cm) with a plaster floor and observed continuously for 60 min. During observation, the following parameters were recorded: the number of encounters between male and female, whether they copulated or not and how often they engaged in copulations. Recording started after observation of the first encounter. To test whether the treatment (methyl geranate versus control) had an effect on the occurrence (yes/no) or number of copulations, we performed a *χ*^2^-test and a Poisson GLM, respectively.

### Effect of breeding partner on methyl geranate emission

This experiment aimed to test the effect of the sex of the breeding partner on methyl geranate emission. Two females were kept together with a male for 48 h, so to that both females were inseminated. Afterwards the females (without the males) were transferred to a new container (20 × 20 × 6 cm) and provided with a large carcass (>30 g) to induce cooperative breeding. A 30 g carcass was prodvided because cooperative same sex brood care usually only occurs on large carcasses^82^. As soon as the first larva had hatched the respective female pair was provided with up to 30 first instar larvae to care for. Additionally, male-female pairs were established and subjected to the same procedure. As in previous experiments, we also provided a large number of *N. vespilloides* pairs with a 10 g mouse carcass to use them as ‘larvae donor' in case a pair did not have enough of their own larvae to add^78^. After 24 h of larval care all females were subjected to individual headspace analyses and then weighed. A Gaussian GLM was used with the sex of the breeding partner as the fixed factor and the amount of methyl geranate emitted as dependent variable. To verify that in the female–female dyads both females performed brood care, we observed their feeding behaviour. In all tested cases, both females were feeding larvae. Furthermore, there was no difference in the weight between females breeding with a female and those breeding a male partner (Gaussian GLM, *N*=28, *F*_1,26_=0.01, *P*=0.92), which is a good indicator that all female had equal access to the carrion resource.

### Headspace analyses

To quantify methyl geranate emission, females were put singly into a glass jar (inner diameter 3 cm) and left undisturbed to accumulate the volatiles emitted by the beetles. Before use, all glasses were silanized with 5% dimethyldichlorosilane solution in toluene to increase their hydrophobicity and prevent adherence of chemical substances. Each glass jar was provided with a moist, Soxhlet-cleaned ball of cotton wool to provide sufficient humidity for the beetles. After an accumulation time of 20 min, air was sucked through the jar at a rate of 200 ml min^−1^ for 5 min using a membrane pump (DC 12/16FK, Fürgut, Aichstetten, Germany). Incoming air was cleaned by an activated charcoal filter (50 mg, Supelco, Bellefonte, PA, USA). The effluent air stream passed through the glass jar and the emitted volatiles were trapped on a thermal desorption filter filled with a mixture of Tenax-TA and Carbotrap as described in ref. 83. For quantification, 20 ng of methyl undecanoate (dissolved in 1 μl methanol) were applied to each adsorbent filter as internal standard. A calibration curve was created by analysing known amounts (5–100 ng) of synthetic methyl geranate that were applied to the adsorbent filter together with 20 ng of the internal standard.

Headspace samples from the first experiment (Methyl geranate and JH profile during a breeding cycle) were thermally desorbed (8 min at 300 °C) using a Shimadzu TD20 automated thermal desorption system connected to a Shimadzu GC 2010 gas chromatograph coupled to a QP2010 plus mass spectrometer. The GC was equipped with a non-polar capillary column (BPX-5, 30 m length, 0.25 mm i.d., 0.25 μm film thickness, SGE Analytical Science, Milton Keynes, UK) and helium was used as carrier gas (50 cm s^−1^ linear velocity). The oven programme started at 50 °C and was raised at a rate of 5 °C min^−1^ to 200 °C, and then at a rate of 15 °C min^−1^ to 280 °C that was held for 10 min. The mass spectrometer was run in electron impact mode (70 eV) and set to a scan range from 35 to 450 *m*/*z*. Headspace samples of all other experiments were analysed using an Agilent Technologies 7820A gas chromatograph equipped with a non-polar capillary column (DB-5, 30 m length, 0.25 mm i.d., 0.25 μm film thickness, J & W Scientific, Folsom, CA, USA) and hydrogen as carrier gas (40 cm s^−1^ linear velocity). Adsorbent filter were introduced via ChromatoProbe kit (CPAV6890, Aviv Analytical Ltd, Israel) and thermally desorbed for 1 min at 310 °C. GC temperature settings were the same as described above.

## Additional information

**Accession codes**: The sequences generated in this study have been deposited in EBI's Short Read Archive (SRA). Raw reads have been deposited in SRA under sample accession codes ERS1048756-ERS1048761. The study is accessible with accession code PRJEB12583 or directly at: http://www.ebi.ac.uk/ena/data/view/PRJEB12583.

**How to cite this article:** Engel, K. C. *et al*. A hormone-related female anti-aphrodisiac signals temporary infertility and causes sexual abstinence to synchronize parental care. *Nat. Commun.* 7:11035 doi: 10.1038/ncomms11035 (2016).

## Supplementary Material

Supplementary Information Supplementary Figures 1-3 and Supplementary Table 1.
