# Supplementary material for: A hormone-related female anti-aphrodisiac signals temporary infertility and causes sexual abstinence to synchronize parental care
Source: Nat Commun. 2016 Mar 22;7:11035. doi: 10.1038/ncomms11035 (PMC4804164; doi:10.1038/ncomms11035)
Supplement: Supplementary Information — Supplementary Figures 1-3 and Supplementary Table 1. [file ncomms11035-s1.pdf]

Supplementary Figures

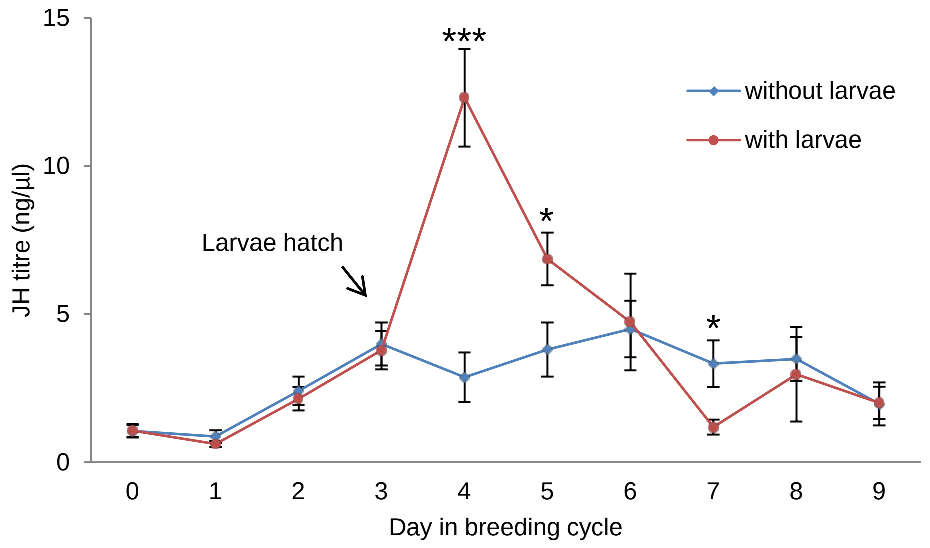

Supplementary Figure 1 | Juvenile hormone III haemolymph titres. JH III titres (mean ± SE) of females during an entire breeding cycle. Females were either allowed to care for their larvae (‘with larvae’, *N* = 184;) or withheld from their larvae upon hatching (‘without larvae’, *N* = 185). There was an interaction effect of treatment group and day (Gaussian GLM: $F_{9,349} = 7.54$, $P < 0.0001$). Females of the treatment group “with larvae” had significantly higher JH III titres on day 4 and 5 than females of the treatment group “without larvae”. Females of the treatment group “without larvae” had significantly higher JH III titres on day 7 than females of the treatment group “with larvae” (* P = 0.05, ** P = 0.01, ***P = 0.001). Note: on day 3 larvae hatched, but had not yet arrived on the carcass.

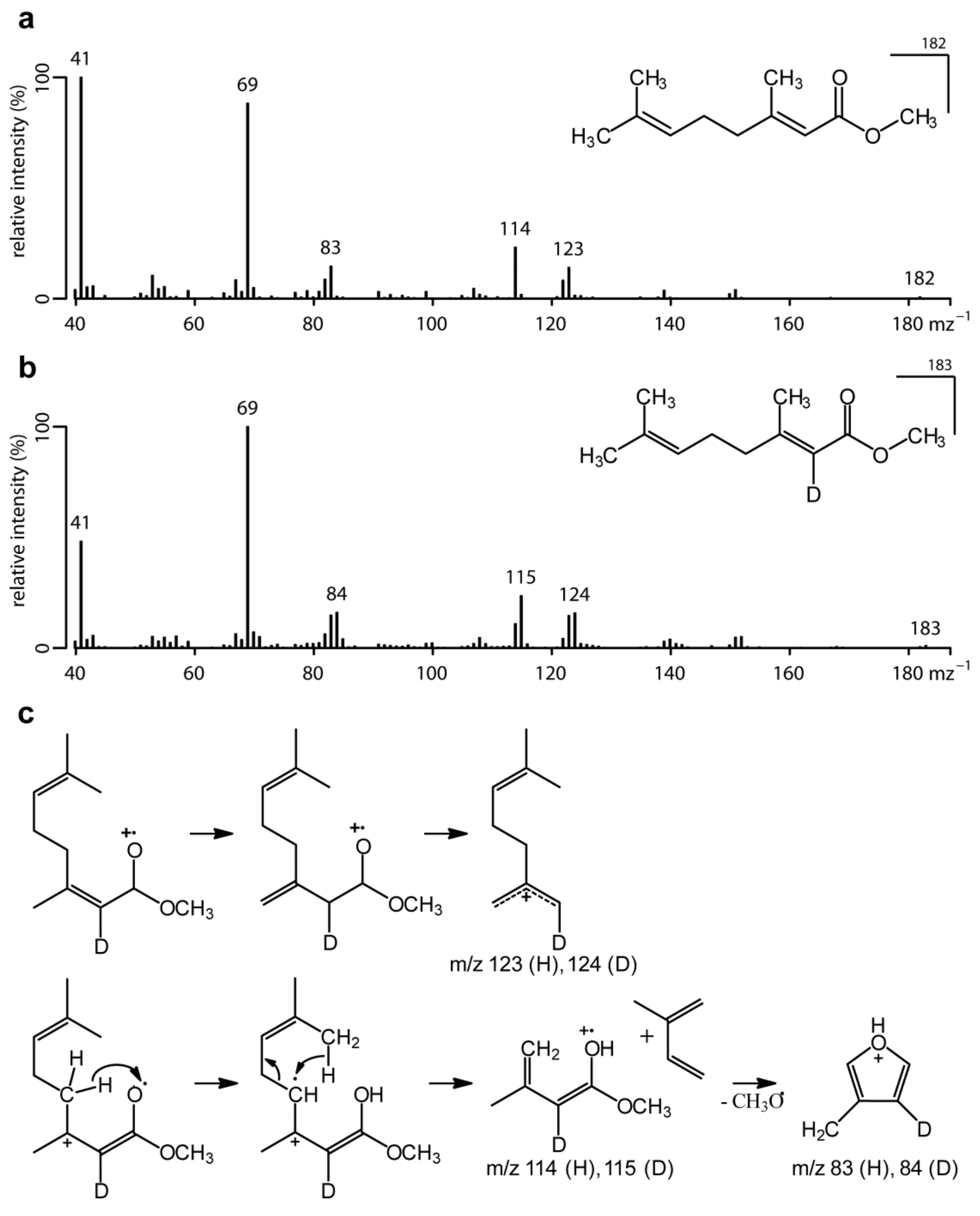

Supplementary Figure 2 | Biosynthesis of methyl geranate. a, b Mass spectra (EI) of methyl geranate released by an untreated *N. vespilloides* female (**a**) and a female injected with the deuterium labelled geranyl pyrophosphate [2-^{2}H]-GPP (**b**). Note the mass shifts for the diagnostic ions m/z 83/84, 114/115, 123/124, and 182/183. Inserts show the structure of methyl geranate without and with the deuterium label, respectively. **c,** Mass spectrometric fragmentation of methyl geranate explaining the diagnostic ions m/z 83, 114, and 123.

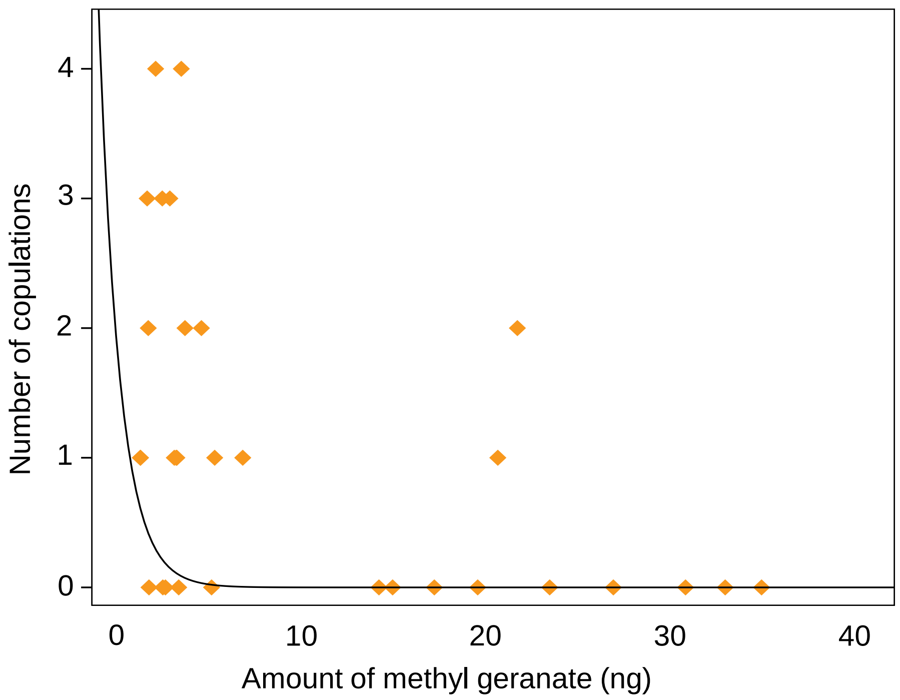

Supplementary Figure 3 | Effect of methyl geranate emission on the number of copulations per female. Females with lower amounts of methyl geranate received more copulations than females with higher amounts of methyl geranate. Symbols represent original data. Curve represents the calculated Poisson distribution. (N = 31, Poisson-GLM: Wald- $\chi^2_{1,29}$ = 7.7, P = 0.006).

Supplementary Table 1. Sample sizes for each subgroup of juvenile hormone III and methyl geranate measurements during an entire breeding cycle shown in Fig. 1b & d. 'MG + JH III' indicates those cases, where both measurements were obtained from the same individual.

	With larvae				Without larvae			
Day	total	JH III	MG	MG + JH III	total	JH III	MG	MG + JH III
0	19	17	16	14	19	19	18	18
1	19	19	19	19	18	18	17	17
2	19	19	18	18	19	19	19	19
3	23	23	18	18	19	18	15	14
4	19	19	17	17	19	19	18	18
5	21	21	16	16	19	18	18	17
6	17	17	15	15	21	20	17	16
7	20	19	18	17	19	18	17	16
8	21	19	18	16	21	21	17	17
9	18	18	15	15	17	16	13	13
total	196	191	170	165	191	186	169	165

Abbreviations: MG = methyl geranate; JH III = juvenile hormone III
